# Next Generation Sequencing: Advances in Characterizing the Methylome 

**DOI:** 10.3390/genes1010143

**Published:** 2010-07-01

**Authors:** Kristen H. Taylor, Huidong Shi, Charles W. Caldwell

**Affiliations:** 1 University of Missouri-Columbia School of Medicine, Ellis Fischel Cancer Center, Columbia, MO 65212, USA; E-Mail: taylorkh@health.missouri.edu; 2 Medical College of Georgia, Augusta, GA 30912, USA; E-Mail: hshi@mcg.edu

**Keywords:** lymphoma, leukemia, next-generation sequencing, methylation, epigenome

## Abstract

Epigenetic modifications play an important role in lymphoid malignancies. This has been evidenced by the large body of work published using microarray technologies to generate methylation profiles for numerous types and subtypes of lymphoma and leukemia. These studies have shown the importance of defining the epigenome so that we can better understand the biology of lymphoma. Recent advances in DNA sequencing technology have transformed the landscape of epigenomic analysis as we now have the ability to characterize the genome-wide distribution of chromatin modifications and DNA methylation using next-generation sequencing. To take full advantage of the throughput of next-generation sequencing, there are many methodologies that have been developed and many more that are currently being developed. Choosing the appropriate methodology is fundamental to the outcome of next-generation sequencing studies. In this review, published technologies and methodologies applicable to studying the methylome are presented. In addition, progress towards defining the methylome in lymphoma is discussed and prospective directions that have been made possible as a result of next-generation sequencing technology. Finally, methodologies are introduced that have not yet been published but that are being explored in the pursuit of defining the lymphoma methylome.

## 1. Introduction

The human genome is comprised of three billion base pairs of DNA, of which ~30 million are CpG dinucleotides, the main sites of DNA methylation. Methylation of cytosine residues at CpG dinucleotides is a prevalent epigenetic modification in mammalian genomes which is known to have profound effects on gene expression [1,2,23]. This epigenetic event occurs globally in the normal genome and is estimated to affect between 70 and 80% of all CpG dinucleotides in human cells [4,5]. These dinucleotides are not uniformly distributed across the genome but occur in clusters such as large repetitive sequences or in CG-rich DNA stretches known as CpG islands (CGIs). The majority of the CGI which are found in intragenic regions, including repetitive sequences such as satellite sequences and centromeric repeats, contain methylated CpG dinucleotides while CGIs which are found preferentially in the promoter regions of genes typically contain unmethylated CpG dinucleotides [6]. Some exceptions to this rule include those CGIs located on the inactive X chromosome in females [7] and those associated with imprinted genes (genes for which only the paternally- or maternally-inherited allele is expressed) which are methylated in the normal state [8,9]. Contrary to normal cells, a number of studies examining DNA methylation in human cancers have shown global hypomethylation and concomitant hypermethylation in the regulatory regions of specific genes [10,11], although this concept has recently been challenged [12]. Methylation of DNA in humans is generally thought to take place via some combination of three DNA methyltransferase enzymes (DNMT1, DNMT3A, and DNMT3B). Whereas DNMT1 is responsible for the maintenance of normal methylation patterns, DNMT3A and 3B are primarily associated with *de novo* methylation. Large-scale studies of DNA methylation have shown that aberrant methylation is a phenomenon present in virtually all types of tumors but it occurs in a generally non-random manner that differs between tumor types. Exactly how the methylation is directed and controlled is still a largely unanswered question. Polycomb group (PcG) proteins are likely to be key in marking certain genes for DNA methylation and in altering histone methylation leading to the formation of repressive chromatin, but the mechanisms by which this occurs are not completely elucidated [13,14,15,16,17,18]. A class of non-coding RNAs, the microRNAs, are also likely modulators of DNA methylation. It has been suggested that miR-143 controls DNMT3A in colorectal cancer [19] and that the miR-29 family regulates DNMT3A and 3B in lung cancer [20]. Furthermore, miR-29b has recently been shown to target all three DNA methyltransferase genes either directly or indirectly in acute myeloid leukemia [21].

**Figure 1:**
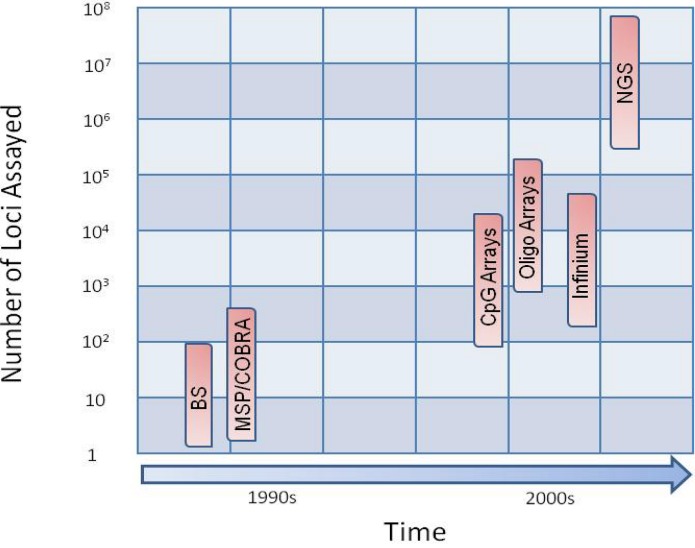
Time *versus* number of loci assayed. The number of loci assayed has increased over time with the development of new technologies. Bisulfite sequencing (BS), methylation specific PCR (MSP) and combined bisulfite restriction analysis (COBRA) were introduced in the 1990s. These methods provided methylation data for up to a few hundred genes in a single experiment. Methylation arrays were introduced in the 2000s and provide methylation data for up to 100s of thousands of loci. (N.B. The latest oligonucleotide array available from Nimblegen allows for the interrogation of more than two million loci). Next-generation sequencing (NGS) was introduced in 2007 and provides single base pair resolution for billions of nucleotides.

A number of methods have been used to examine DNA methylation at whole- or sub-genome scale, and over time these methods have progressed from the ability to characterize the methylation profiles of candidate genes to the ability to develop genome-wide methylation profiles (Figure 1). These have been discussed in detail in an excellent review by Peter Laird [22]. Generally, these can be categorized into the preparative method and the analytical or detection systems employed. The preparative methods typically fall into three broad classes that include enzyme digestions, affinity enrichments, or treatment with sodium bisulfite while the detection systems can be described as locus-specific, gel-based, array-based, or as discussed below, next-generation sequencing (NGS) based methods. Although none of these earlier methods is perfect, each has allowed contributions of valuable epigenetic data to the scientific community from a large number of laboratories. In the advent of next generation sequencing, we now have the ability to detect aberrant epigenetic events in cancer at high resolution. This review describes the technologies and methods currently being used to develop high resolution methylation profiles and the progress that has been made using NGS to characterize the methylome in lymphoma.

## 2. Cutting-edge Technological Advances

In a relatively short time period, since its inception in 2007, the power of NGS technology has transformed the science of genomic research. Currently, five commercial NGS platforms are available; however, Roche/454 (Branford, CT), Illumina/Solexa (San Diego, CA), and Applied Biosystems/SOLiD^TM^ (Carlsbad, CA), dominate the sequencing market shares. Additional platforms such as Pacific Biosciences (Menlo Park, CA), are on the horizon. A feature that all NGS platforms share is the parallel sequencing of clonally amplified or single DNA molecules that are immobilized on an array or flow cell. This feature fundamentally sets NGS apart from conventional capillary-based sequencing in that millions of sequence reads can be generated in parallel rather than 96 at a time. The significant increase in throughput has lead to a dramatic reduction in sequencing cost and a massive accumulation of genomic sequences. The Human Genome Project which was completed in 2003 by Sanger sequencing took 13 years to complete and cost approximately $2.7 trillion dollars. Now, in 2010, a human genome can be sequenced in eight days with 30x coverage for approximately $10,000 dollars using the latest HiSeq2000 sequencer from Illumina. The NGS platforms have facilitated many novel sequencing applications, particularly in the area of epigenomic research. For example, in 2009, the first human epigenome was sequenced using NGS [23]. Although all NGS platforms share some common features, they also differ significantly. (See [24] for an in depth review of the sequencing technologies and a comparison of current specifications). These differences will affect the applications of each platform and the challenges of bioinformatic analysis as described below.

### 2.1. Roche (454) genome sequencer FLX

The 454 Genome Sequencer FLX (454 GS FLX) was the first commercially available NGS to combine single-molecule emulsion PCR with massively parallel pyrosequencing. The sequencing chemistry is based on the principle of pyrosequencing in which a pyrophosphate molecule is released upon nucleotide incorporation by DNA polymerase for luminescence detection. The clonal** amplification of sequencing templates bound on agarose beads is achieved by emulsion PCR which uses a vigorously mixed oil and aqueous mixture to create water-in-oil microvesicles. These microvesicles serve as micro-bioreactors for the PCR reaction. Under limiting-dilution conditions only one bead will be present in each water-in-oil vesicle. Each agarose bead surface contains several million copies of the same single stranded sequencing template, while millions of such beads can be generated in a matter of hours [25]. The microbeads are enriched and deposited into 3.4 million picoliter-scale sequencing reaction wells and combined with sequencing chemistry (See http://www.454.com for an explanation of the workflow and technology). Each picotiter plate can be divided into 16 portions allowing for the simultaneous sequencing of 2, 4, 8 or 16 samples. Over one hundred bar-coded sequencing adaptors are available and therefore up to a thousand samples can be multiplexed on one picotiter plate. The biggest advantage of 454’s sequencing technology is the read length which is on average 450 bp using the newest “titanium” version compared to 50 to 125 bp read lengths generated by the other two major platforms. The longer reads facilitate *de novo* assembly for certain sequencing applications. About 1GB of data from one million sequencing reads can be generated in a single seven hour run on the 454 GS FLX as opposed to a multiple day run on both the Illumina GA and ABI SOLiD^TM^ platforms. However, the Illumina GA, and ABI SOLiD^TM^ sequencers produce approximately 100 million reads per run. Therefore, the sequencing cost per base is higher with the 454 GS FLX. The 454 GS FLX was also the first NGS platform to be used for epigenetic studies. The 454 website currently lists 23 publications in the field of epigenetics and of these four used bisulfite sequencing to determine the precise location of CpG methylation in cancer [26,27,28,29]. One major limitation of the 454 technology is that it is difficult to correctly call homopolymers greater than 3-4 bases in length. This limitation is exasperated in bisulfite sequencing because in many GC rich regions, a long stretch of poly-A or poly-T will appear due to the conversion of unmethylated C to T. However, since the bisulfite sequence reads are not assembled but merely aligned to the reference genome sequence, modifications to the mapping algorithms can solve this problem. In addition, the longer sequencing reads produced by this technology greatly enhances the opportunity for accurate alignment of the bisulfite converted sequences to the reference genome. Approximately 70% of bisulfite sequencing reads generated by the 454 GS FLX can be mapped accurately to the reference genome while there is significant variation reported in the mapping rates using the Illumina GA and SOLiD^TM^ platforms due to the shorter read lengths produced by these technologies [30,31,32].

### 2.2. Illumina genome analyzer

The Illumina GA was the first short read NGS platform based on the concept of “sequence-by-synthesis” (SBS). It produces sequence reads of 36 to 125 bp from tens of millions of clonally amplified DNA fragments on a flow cell. The Illumina technology uses a novel PCR process known as bridge PCR to achieve clonal amplification of sequencing templates on the surface of a glass flow cell comprised of eight separate lanes. Each lane has covalently attached oligonucleotides that are complimentary to specific adaptors that are ligated to the library fragments. Under limiting-dilution conditions, adaptor-modified single-stranded template DNA is hybridized to the anchor oligonucleotides on the flow cell followed by subsequent cyclic PCR to convert a single DNA molecule into a clonally amplified cluster consisting of approximately 1000 molecules. The Illumina sequencing chemistry uses four different fluorescent dye labeled nucleotides and resembles traditional Sanger sequencing. However, these nucleotides are modified at the 3’-OH group to ensure that only a single nucleotide is incorporated in each sequencing cycle. Each sequencing cycle is followed by an imaging step to capture the incorporated nucleotide at each cluster. The fluorescent group is removed after imaging and the 3’-end is re-opened for the next base incorporation cycle. Up to 125 cycles can be repeated using existing protocols which generate up to 125 bp sequence reads due to the improvement of sequencing chemistry and image analysis software. A typical run yields approximately 70-100 million reads and takes three to six days depending on the read length desired. Because of its robust performance and the improvement of read length and accuracy over time, the Illumina GA occupies over 60% of the current market. Many different applications have been developed on this platform including RNA-seq, small RNA sequencing, ChIP-seq, and whole genome and targeted sequencing. The Illumina GA has been used for shotgun whole-genome bisulfite sequencing of human, mouse and plant genomes [23,33,34,35]. Also, a method known as targeted capture bisulfite sequencing was developed for the Illumina GA instrument [30,32,36]. In total there are more than 40 epigenetics publications using the Illumina GA instrument. Of these, 17 address DNA methylation and two address DNA methylation in cancer [32,37]. Many early bisulfite sequencing experiments produced less than 30% mappable reads compared to ~ 80-90% mappable reads for non bisulfite-treated sequencing using the same short read alignment software [30,32,36]. This discord in mappability is due in large part to the limitations of current mapping algorithms to align bisulfite converted sequences. Furthermore, because the mapping rate may vary significantly between samples, the comparability of data may be jeopardized.

### 2.3. Applied biosystems (SOLiD^TM^)

The SOLiD^TM^ (Sequencing by Oligo Ligation and Detection) system was released in 2007 by Applied Biosystems and is based on a unique sequencing process called “sequencing by ligation”. The approach was initially developed by George Church’s group at Harvard and was termed polony sequencing [38]. Applied Biosystems refined the technology and developed a two color sequencing chemistry for this instrument. Similar to the 454 system, the SOLiD^TM^ system also uses emulsion PCR to generate clonally amplified sequencing templates. However, much smaller magnetic beads (1 um) are used in the emulsion PCR process and the beads are covalently attached to the surface of glass slides. With the current SOLiD^TM ^4 system, over 700 million reads per run can be achieved for a single read library. Each run requires 6-8 days and produces 40-50 GB of data with a read length of 50 bp. In addition to genome sequencing, RNA-seq, small RNA sequencing, and ChIP-seq, bisulfite sequencing of a bacterial genome has recently been published using this technology [39]. The main advantages of this technology are increased throughput and increased sequencing accuracy compared to the other sequencing platforms. 

### 2.4. Other sequencing technologies.

Helicose is an alternative NGS platform which uses a similar principle to the three major platforms but does not rely on clonal amplification. It is the only platform to date that has truly achieved single molecule parallel sequencing. However, because of its late entry into the market, and a relatively high cost, sales have considerably lagged behind the three platforms described above. In addition, the Polonator has been commercialized by Dover systems and is based on the original polony sequencing methods developed by Church’s group [38]. This system is relatively inexpensive but the throughput and read lengths are lower than the major commercial platforms; therefore, few laboratories are using this system. Complete Genomics, a company based in Mountain View, California, has also published a sequencing method [40]. Speed and cost have been Complete Genomics' key selling points. They claim that by June of 2010 the cost of materials will be approximately $1,000 per genome.

### 2.5. Third generation sequencing technologies.

In addition to the second generation sequencing platforms mentioned above, third generation sequencing platforms are beginning to emerge. For example, Pacific Biosciences uses a single-molecule technology with engineered DNA polymerase to capture and record the incorporation of fluorescently labeled nucleotides into growing complementary nucleic acid strands. While this technology produces fewer reads than 454’s GS FLX, Illumina’s GA and Applied Biosystem’s SOLiD^TM^, it is distinguished by its very long reads and ultra-fast real time sequencing. This transformative technology will enable a new paradigm in genomic analysis. Particularly interesting, the tempo and duration of the resulting fluorescent pulses yield a rich set of information about polymerase kinetics and allow direct detection of various forms of modified nucleotides, including 5-methylcytosine, 5-hydroxymethylcytosine, and N6-methyladenosine. Therefore, it is possible to detect methylated bases without the need for bisulfite conversion of genomic DNA. Unlike current bisulfite-sequencing techniques which are limited by short read lengths and by a reduction in genomic complexity, the Pacific Biosciences method will enable mapping of methylation patterns even within highly repetitive genomic regions. 

## 3. Methylome Methodologies

Genome-wide methylation methodologies can be divided into those that divulge the precise location of methylated CpG dinucleotides (site specific) and those that disclose methylated regions of the genome. The site specific methods rely on bisulfite conversion of DNA while the regional methods rely on enzyme digestion or the affinity of either antibodies or proteins to methylated DNA. Sodium bisulfite converts unmethylated cytosines to uracils but has no effect on methylated cytosines. This conversion can then be exploited in a sequencing context because cytosines that were originally unmethylated will appear as thymines after amplification and those that were methylated will remain cytosines. The net effect of this conversion process is that the complexity of the genome is greatly reduced since consequently the genome now consists of three bases instead of four with the exception of methylated cytosines. Bias may be introduced in site specific methods due to incomplete bisulfite conversion and the efficiency of bisulfite PCR. These methods also require extensive bioinformatics for aligning the resulting sequences to the bisulfite converted genome and therefore may not be a suitable option for laboratories that do not have bioinformatic support. 

Alternately, affinity based methods enrich for the methylated fraction of the genome by “pulling down” methylated DNA fragments. These methods are limited by the sensitivity and specificity of the antibody or binding protein employed. In general, these methods are more effective in enriching for regions of the genome with a high density of methylation and less efficient in CpG poor regions of the genome. Given that highly repetitive sequences are densely methylated, a considerable proportion of the sequences generated may be impossible to unambiguously align to the genome. In addition, digestion with methylation sensitive or methylation dependent enzymes enrich for the unmethylated or methylated regions of the genome respectively. When combined with NGS fragments that are ‘missing’ after digestion with a methylation sensitive enzyme represent the methylated regions of the genome while those that are present after digestion with a methylation dependent enzyme represent the methylated regions of the genome. Because the affinity and restriction enzyme methods confer only regional methylation information, additional experiments are needed to identify the precise location of CpG methylation within these regions.

### 3.1. Site specific 

*Whole-genome bisulfite sequencing* provides coverage at single base pair resolution and is therefore the most comprehensive of the site specific methodologies. In this method the DNA is sheared and bisulfite adaptors are ligated to the fragments. These adaptors can be synthesized with 5-methylcytosine so that the cytosines remain cytosines after bisulfite conversion or alternatively one can use modified amplification primers to include the bisulfite converted version of the adaptor. It is important to note that the sequence of the adaptors after bisulfite conversion must be complimentary to the seeding oligos. Therefore, it is imperative that the cytosines are preserved after conversion in order for the fragments to be clonally amplified on a flow cell. After the adaptors are added, the sample is bisulfite converted and the converted fragments are PCR amplified. These amplified fragments are then used in the library preparation for high throughput sequencing. In 2008, Cokus and colleagues published the complete methylome of Arabidopsis using this technique coupled with the Illumina GA sequencing platform [33]. This technology produced 3.8 billion high quality nucleotides providing 20X coverage of the 120 Mb Arabidopsis genome. Of these, 2.6 billion nucleotides mapped uniquely to the genome and covered 93% of all cytosines that could be theoretically covered. The size of the Arabidopsis genome makes it amenable to this type of study, however, the size of the human genome is approximately 150X larger than the Arabidopsis genome and therefore performing these experiments in human (or other mammals for that matter) is cost prohibitive at this time. Nonetheless, this technology was used by Laurent and colleagues to compare human cell types at three progressive stages of differentiation [41] and by Lister and colleagues to compare human embryonic stem cells and fetal fibroblasts [23]. To date, the only other group to use the shot-gun bisulfite sequencing approach constructed an *Escherichia coli* methylome using the SOLiD^TM^ platform [39]. Whole-genome bisulfite sequencing provides unbiased coverage of the genome allowing for the interrogation of regions of the genome that are often missed using other methodologies.

While bisulfite treatment followed by shotgun sequencing remains a daunting task for large genomes, there are methods available that enrich CpG fragments which greatly reduces the depth of sequencing required for whole-genome coverage. Some of these methods are technically challenging and each has unique biases that are introduced. Reduced representation bisulfite sequencing, targeted capture and padlock probes are described in the following sections. 

**Figure 2:**
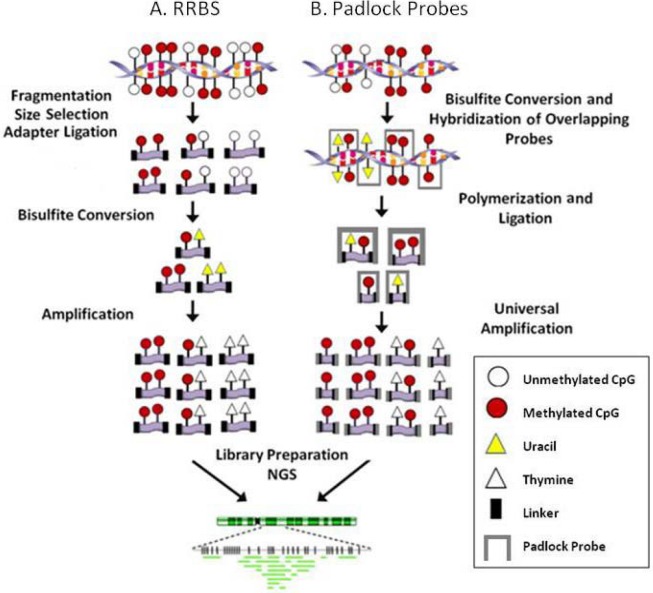
Site specific NGS methodologies. **A.** Reduced representation bisulfite sequencing (RRBS). **B.** Padlock probe assisted multiplex amplification. NGS: Next generation sequencing.

*Reduced representation bisulfite sequencing* (RRBS) enriches for CpG rich regions of the genome without the need for a known set of targets (*i.e.*, candidate genes, CpG islands, promoters, enhancers *etc.*). In this method DNA is fragmented with a restriction enzyme (or combination of enzymes), adaptors are ligated to size selected fragments then bisulfite treated and amplified to produce a library for NGS (Figure 2A). Enrichment using restriction enzymes is not truly genome-wide but includes a large portion of the genome and has the advantage of targeting non-repetitive CpG containing regions of the genome. The data generated includes regions of the genome that are in close proximity to a specific restriction enzyme’s recognition sequence. This is helpful in regards to the bioinformatic analysis of these data because the genome is reduced and therefore less computational power is required to align the sequences. In choosing the appropriate enzyme to use to reduce the genome to be sequenced, one must consider which NGS platform will be used since each sequencing platform has unique properties. For example the Illumina and Applied Biosystems platforms produce short sequence reads and therefore the size of the restriction fragment produced should remain small while 454 can handle longer fragments and to reach its full potential restriction enzymes that result in larger fragments should be chosen in order to cover as much of the genome as possible. 

Meissner and colleagues first published the concept of RRBS in 2005 [42]. After the restriction of mouse DNA with BglII (recognition sequence AGATCT), fragments between 500 and 600 bp were size selected and then adaptors were ligated to the size selected fragments. The fragments were then subjected to bisulfite treatment, amplified using bisulfite-modified adaptor sequences, cloned into a plasmid vector and then sequenced using conventional Sanger sequencing. To align the data, the generated sequences were searched against a reduced representation database of *in silico* size selected and bisulfite treated BglII fragments. This method was modified in 2009 by Meissner’s group for amenability to the Illumina sequencing platform [43]. In this protocol MspI, a methylation insensitive enzyme, was used to digest mouse DNA. The use of this particular enzyme ensures that at least two data points will be produced for each restriction fragment since the MspI recognition sequence is CCGG. The resulting fragments were end repaired and then methylated adaptor sequences were ligated to the fragments. The adaptor-ligated fragments were size selected for 40-120 bp and 120-220 bp and two sequencing libraries were produced. The selected fragments were bisulfite converted and then amplified to produce two sequencing libraries instead of a single library to reduce size related amplification bias. 

In addition to using single enzymes for RRBS, combinations of enzymes can also be used. Zeshnigk and colleagues used MseI (TTAA) and Tsp509I (AATT) to deplete AT rich regions of the genome in combination with NlaIII (CATG) and HpyCH4 (TGCA) [44]. After digestion, adaptors were ligated to blunt-ended and phosphorylated fragments. The adaptor-ligated fragments were treated with bisulfite, amplified and then size selected (440-900 bp) before sequencing using the 454 GS FLX. This study compared the methylation present in human female white blood cells with the methylation present in human sperm. Theoretically this combination of enzymes should produce data that covers 12,482 CGI (~45% of annotated CGI); however, only 21-22% of annotated CGI were covered and only 7.6% and 7.9% of the female white blood cell and sperm cell reads respectively mapped to a CGI. While this may be an effective enrichment strategy, these results suggest that this library preparation method is biased toward non-CG rich regions; however, it may be possible to increase the CGI coverage by obtaining more sequence reads. 

*Targeted capture* microarrays comprised of probes that encompass regions of interest or targeted probe libraries within solution can be used to enrich for regions of the genome before sequencing. Targeted probes can be developed to capture bisulfite converted or non-converted sequences. An important consideration, if the bisulfite converted genome is the targeted genome, is that capture probes must be designed such that all permutations after bisulfite conversion are included. A clear advantage to this method is that it provides a reduced genome without the sequence constraint inherent in RRBS which utilizes restriction enzymes. In 2009 Hodges and colleagues published a method for targeting bisulfite converted DNA to enrich for regions of interest in the genome [32]. In this method, genomic DNA was sonicated and then methylated Illumina adaptors were ligated to the fragments. The fragments were then size selected (200-300 bp) and bisulfite converted. The converted templates were PCR amplified and captured on a custom Agilent 244 k array. The captured fragments were then eluted and sequenced using the Illumina GA platform. These authors sequenced a normal and a cancer cell line and report an enrichment of over 700 fold in the normal cell line and over 1300 fold in the cancer cell line. In both cell lines more than 90% of the target region was covered by at least 10 sequencing reads. However, only 6.43% of the total reads in the normal cell line and 11.98% of the total reads in the cancer cell line mapped to the targeted region. Therefore, a disadvantage of this method is that the capture is often imprecise and fairly inefficient. On the other hand, because an amplification step is required in this protocol, less DNA is required as starting material than is needed for arrays targeted at non-bisulfite converted DNA.

*Padlock probe assisted multiplex amplification* is performed in solution and has the capability of targeting more of the genome than the current array based capture platforms. Padlock probes are designed such that a region of interest is covered by overlapping probes on both the sense and antisense DNA strand (Figure 2B). Each probe consists of two target specific arms connected by a universal backbone sequence and hybridizes to the DNA in two places leaving the backbone sequence unbound. The gap between the two arms is polymerized and then ligated to form circular DNA. The backbone sequence is then used for universal PCR allowing for the amplification of tens of thousands of probes within a single reaction. Extensive bioinformatics is required for the design of the padlock probes because each probe must be normalized for melting temperature and length. Recently, two groups utilized this technology for methylation analysis. In each case, a library of padlock probes was annealed to bisulfite converted DNA. Next the 3’ ends were extended and ligated to the 5’ ends. The linear DNA was removed by exonucleases and then the circularized DNA was amplified with common primers. Deng and colleagues developed padlock probes to capture 175-225 bp regions including CpG islands, promoters and the transcription start site of genes involved in development or pluripotency [30]. In total 10,582 probes were required to cover 2.1 Mbp of the genome. However, since bisulfite conversion results in all unmethylated cytosines being converted to uracils, 30,000 probes were developed in which all C/T combinations were included for those probes that contained CpG sites. They observed a 10,000 fold difference in capture efficiencies between the most and the least efficient probes. Therefore, some probes required re-synthesis (to gain efficiency) and some probes required the addition of suppressor oligos (to reduce efficiency) which is the primary shortcoming of this method. Ball and colleagues focused on the ENCODE pilot project regions and avoided CpGs in the hybridizing arms so that only one probe was necessary per locus [36]. Their design targeted regions of the genome that are not associated with CGI and therefore provide data from regions not typically included in methylation studies.

### 3.2. Regional

*Restriction-enzyme methods* create a genome-wide methylation profile for regions of the genome that are encompassed by DNA fragments created after digestion with either a methylation sensitive or methylation dependent enzyme (or combination of enzymes). Ball and colleagues utilized the methylation sensitive enzyme HpaII to create a library of tag fragments that were then sequenced using the Illumina platform [45]. In this method, a library of tag fragments from all restriction fragments was created and then subjected to NGS. The methylation of a particular fragment was inferred based on the number of times it was observed. For example, fragments that were observed many times had low levels of methylation while those that were not observed had high levels of methylation. As a control, the authors also created an MspI library which has the same recognition sequence as HpaII but cuts either methylated or unmethylated CCGG sequences. After final analysis, the authors concluded that the control library was not necessary because the results from the HpaII library alone was highly correlated with the methylation present at individual sites. This method is better suited for distinguishing between highly and moderately methylated fragments as opposed to moderately and weakly methylated fragments because there is more noise associated with the weakly methylated (and therefore more highly digested) fragments. Concurrently, a second group published a modification of the HpaII tiny fragment enrichment ligation mediated PCR (HELP) protocol, HELP-seq [46]. For this method, the authors removed the adaptors from the LM-PCR products with MspI and then used the fragments to create sequencing libraries. The results of HELP-seq and HELP with microarray were compared and found to be strongly concordant. However, HELP-seq identified more hypomethylated loci and allowed the identification of shorter sequences that were not able to be represented on the microarray. In 2009, Wang and colleagues used a methylation dependent enzyme McrBC to identify methylation in the maize genome [47]. Cleavage with McrBC requires the presence of two methylated recognition sequences separated by 40-3000 base pairs. In this method, genomic DNA was digested with McrBC and then size selected fragments were used for library construction and then sequenced using the Illumina platform. Restriction based methods provide an excellent initial screen of the genome but are biased toward regions of the genome that contain restriction enzyme recognition sequences and require additional experiments to determine the precise location of methylated cytosines within the methylated fragments. 

**Figure 3:**
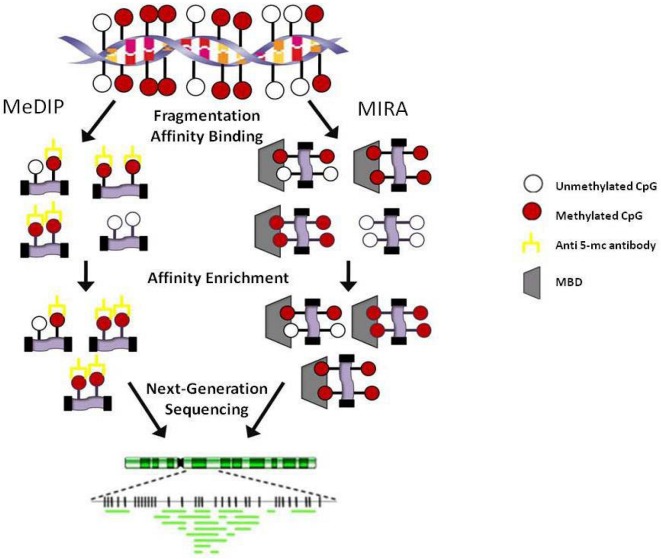
Affinity-based methodologies. MeDIP: Methylated DNA immunoprecipitation; MIRA: Methylated CGI recovery assay; MBD: Methyl-binding domain.

*Affinity methods* such as methylated DNA immunoprecipitation (MeDIP) and methylated CpG island recovery assay (MIRA) do not rely on the bisulfite modification of DNA [48,49]. MeDIP employs an antibody directed against 5-methylcytosine while MIRA (and modifications of MIRA) utilizes methyl-binding proteins (Figure 3). One major difference between the methods is that MeDIP requires DNA to be single-stranded in order to achieve efficient pull down which is sometimes difficult to achieve in regions of high CpG content and MIRA does not. Both MeDIP and MIRA can now be performed using commercial kits available from multiple companies. Briefly, for MeDIP, the DNA is sheared into 300-1 kb fragments, denatured and incubated with anti-5 methyl-cytosine. 

Magnetic or agarose (sepharose) beads are added and the methylated DNA is separated from the unmethylated DNA. Recently, Butcher and Beck published a modified version of this protocol amenable to high-throughput methylome analysis termed AutoMeDIP-seq [50]. In MIRA, a His-tagged MBD2b/MBD3L1 complex is incubated with DNA fragmented either by shearing or by digestion with MseI. The methylated protein-DNA complexes are captured using magnetic beads and the DNA is eluted while the protein complexes are degraded. A modification of this technique was recently used to characterize the methylome of three cancer cell lines and termed methyl binding domain (MBD) isolated genome sequencing [51]. Bioinformatic analysis of data generated after affinity enrichment is complicated by the need to take into consideration the density of CpG dinucleotides in a given region and by the inherent enrichment of repeat regions which can be difficult or even impossible to align to the genome. Overall, affinity methods provide a genome-wide assessment of the methylation present but do not give information on specific CpG dinucleotides and are biased toward CG rich regions of the genome. 

## 4. Progress Toward Characterizing the Methylome of Lymphoma and Leukemia 

### 4.1. Genome-wide microarrays

Genome-wide methylation studies in lymphomas have progressed rapidly in light of the development of genome-wide technologies. Some of the earlier microarray platforms allowed for the investigation of DNA methylation present within select CpG islands [52,53]. The coverage of these arrays was limited but facilitated the discovery of putative tumor suppressor genes that are methylated in lymphomas and leukemias. Together with the identification of aberrantly methylated genes, investigators were able to differentiate between tumor types and subtypes [54,55,56,57,58,59] and to identify methylation hotspots in the genome [56,60]. The lessons learned from these studies prompted the development of commercial arrays that cover CpG islands, promoters of genes and even the whole genome (Agilent, Nimblegen Affymetrix, Illumina). These comprehensive arrays have not only allowed investigators to continue to distinguish between disease types, subtypes and outcomes [61,62,63,64,65,66,67,68,69] but have also allowed for the identification of enriched gene pathways and functional groups including PRC2 target genes [65,70,71] which has led to a better understanding of the biology of lymphomas and leukemias. These discoveries have profound implications with regards to prognosis, diagnosis and possibly even treatment of lymphomas and leukemias and highlight the importance of fully elucidating epigenomic changes in these disease types. 

### 4.2. Ultradeep bisulfite NGS of amplicons in B-cell lymphomas

**Figure 4:**
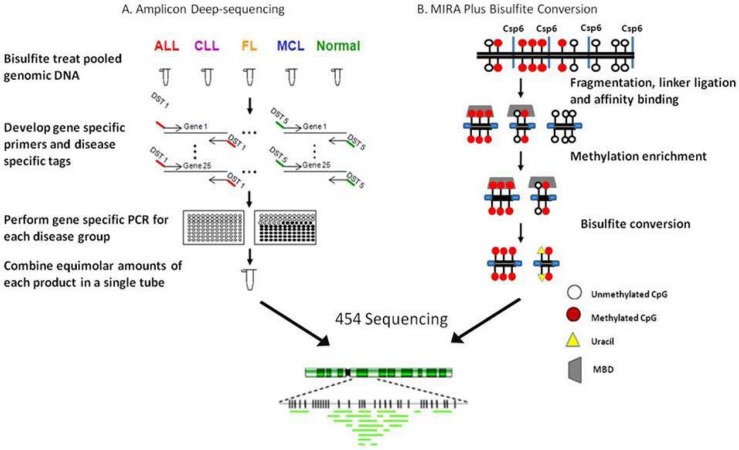
NGS approaches used to study lymphoid malignancies. **A.** Amplicon deep-sequencing. ALL: acute lymphoblastic leukemia; CLL: chronic lymphocytic leukemia; FL: follicular lymphoma; MCL: mantle cell lymphoma; DST: disease specific tag. **B.** MIRA plus bisulfite conversion.

In 2007 our group developed a novel approach that allowed the simultaneous detection of methylation present in the CpG rich region of 25 genes in over 40 cases of primary cells [28]. To accomplish this goal, primer sets with disease specific tags on the 5’ end were developed so that after NGS the groups could be identified (Figure 4A). Five groups were included, normal peripheral blood mononuclear cells (PBMCs), acute lymphoblastic leukemia (ALL), chronic lymphocytic leukemia (CLL), follicular lymphoma (FL) and mantle cell lymphoma (MCL). Ten individual samples within the same group were pooled together and then bisulfite treated. Gene specific primers containing disease specific tags were used to generate 25 amplicons for each group. Using clustering algorithms, differential methylation between diseases was shown. While this was not a genome-wide study it allowed for deep sequencing of amplicons representing genes whose aberrant methylation was previously shown to be methylated in lymphomas or other cancers [55,56,72,73]. Each amplicon was sequenced 100-1000 times providing improved statistical power over that of standard bisulfite sequencing. Twenty of the 25 amplicons studied showed an increase in methylation in various diseases as compared to PBMCs. Quantitative differences in methylation between diseases were also identified and there was a significant increase in the methylation of the 25 amplicons in ALL and FL samples compared to CLL and MCL. Unlike methods which provide regional methylation information, this method identified the precise location of the methylation present within the promoters of a subset of genes. A spreading of methylation was observed from the periphery towards the center of select CpG islands in ALL and FL samples. In addition to generating methylation profiles, deep bisulfite sequencing of amplicons allowed for the determination of an association between a SNP and methylation and also provided mechanistic insight with regards to the spreading of methylation. 

### 4.3. RRBS/MIRA plus bisulfite sequencing in a follicular lymphoma cell line:

In addition to the published work described above, more recently we developed an approach integrating the concept of RRBS with MIRA plus bisulfite conversion to produce a library that was sequenced using the 454 GS FLX sequencer (Figure 4B). The inclusion of the enrichment step before bisulfite conversion allows for the targeting of methylated regions of the genome but is biased toward GC rich DNA sequences. Briefly, genomic DNA was fragmented using Csp6I and then ligated to adaptors, methylated adaptor-ligated fragments were enriched using a methyl-binding protein and the enriched fragments were subjected to bisulfite modification. A sequencing library was constructed and the follicular lymphoma cell line, RL, was sequenced. The genome-wide methylation patterns were compared with gene expression and histone modification profiles from the same cell line. More than 11,000 methylated regions of interest (MRI) were identified covering 4033 CpG islands. Methylation was detected in members of the HOX clustered genes, the protocadherin cluster, SOX genes and Frizzled protein genes and there was a significant overlap in methylation and PRC2 target genes. Genome-wide H3K4Me3, H3K27me3 and SUZ12 binding profiles were generated to compare to the MRIs identified by NGS. As expected, there was no significant overlap of methylation with H3K4 trimethylation. There was also no significant overlap of methylation and H3K27 trimethylation or SUZ12 binding *in vivo*. Furthermore, a SNP in the HLA-A gene cluster was associated with methylation status of 2 neighboring CpG sites. The longer bisulfite sequences obtained by 454 sequencing made sequence alignment accurate and reliable and allowed us to confirm a large number of methylated genes in RL cells and to identify distinguishing methylation patterns in the promoters of these genes. 

**Figure 5:**
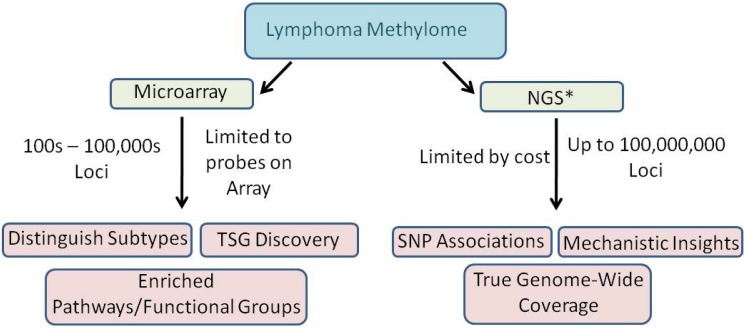
Microarray *versus* NGS in lymphoma methylation studies. NGS: Next-generation sequencing; TSG: tumor suppressor gene; SNP: single nucleotide polymorphism. * NGS includes all outcomes of microarray in addition to the outcomes presented in the diagram.

We are at the forefront of exploiting NGS technologies to characterize the methylomes of lymphoid malignancies. The lessons learned and the information amassed from methylation microarrays is astounding but progress in this endeavor will increase exponentially now that we have the ability to produce true genome-scale data sets (Figure 5). The NGS technologies afford the opportunity to characterize the methylome much more deeply which is paramount to fully elucidating the mechanisms associated with, and the impacts of, aberrant methylation in lymphoid malignancies. 

## 5. Beyond the Methylome

While DNA methylation was the focus of this review, there are other important epigenetic alterations which are also receiving deserving attention. For example, in normal cells, histone acetyltransferases (HATs) add acetyl groups to residues on histone tails that leads to a relaxation of the chromatin structure, allowing gene transcription to occur. HAT activity is counteracted by histone deacetylases (HDACs), enzymes that remove the acetyl groups leading to compaction of nucleosomes and transcriptional downregulation. Inhibitors of HDACs (HDACi) are thought to maintain chromatin in an open structure and are therefore potential pharmacologic agents because many tumors overexpress HDACs and this results in suppression of gene transcription (reviewed in [74]). A number of genome-wide studies have identified combinatorial patterns of histone modifications that may correlate with gene structures and functions [75,76,77,78]. These studies have mainly addressed the processes of acetylation or methylation at lysine (K) or arginine (R) residues in histone protein tails. Generally, trimethylation of histone H3K4 is associated with active transcription, while that of H3K27 is most commonly associated with downregulation, although both marks can appear simultaneously on bivalent gene promoters that may be poised for rapid expression or downregulation.

Certain epigenetic modifications of chromatin by protein arginine methyltransferases (PRMTs) are also crucial for normal cell growth and differentiation. The mechanisms controlling this epigenetic process are not as well understood compared to lysine methylation. The SWI/SNF-associated protein PRMT5 transcriptionally represses its target genes by methylating histone 3 at arginine 8 (H3R8) and histone 4 at arginine 3 (H4R3) [79]. PRMT5 protein levels are increased in CLL cell lines secondary to the altered expression of PRMT5-specific miRs 19a, 25, 32, 92, 92b, and 96 (discussed below) that results in an increase of global symmetric methylation of H3R8 and H4R3. An evaluation of both epigenetic marks at PRMT5 target genes such as cell cycle pocket proteins RB1 (p105), RBL1 (p107), and RBL2 (p130) showed that at their promoters H3R8 and H4R3 are hypermethylated, which then leads to transcriptional repression. Using the human beta-globin locus as a model, symmetric dimethylation of H4R3me2 by PRMT5 is required for subsequent DNA methylation [79]. H4R3me2 is a direct binding target for DNMT3A, which interacts through the ADD domain containing the PHD motif. Loss of the H4R3me2 mark leads to reduced DNMT3A binding, loss of DNA methylation and gene activation. Thus, it was proposed that DNMT3A functions as both a reader and a writer of repressive epigenetic marks at this locus, thereby directly linking histone and DNA methylation in gene silencing.

Small RNA molecules, referred to as microRNA or miR, act as translational repressors of many mRNAs and each may affect hundreds of genes [80]. MiRs are processed after transcription by Drosha and Dicer enzymes and ultimately associate with an RNA-induced silencing complex (RISC) that binds target mRNAs through partially complementary sequences that then reduce their translation or stability. Human miR genes are distributed throughout the genome in a non-random manner and are frequently located in chromosomal fragile sites and cancer-associated genomic regions where they may act as molecular switches (reviewed in [81,82]). As with other coding genes, miR genes have specific functions, and are also susceptible to epigenetic modifications such as DNA hyper- and hypomethylation [83,84]. Some miRs play a fairly direct role in chromatin modification by targeting post-transcriptional regulation of key enzymes. For instance, the miR-29 family targets the three main DNA methyltransferases of humans either directly or indirectly in lung cancer and acute myeloid leukemias [20,21]. These miRs directly target the 3’UTR of DNMT3A and DNMT3B, but affect DNMT1 indirectly by targeting SP1 which functions as a transactivator of this enzyme gene. These enzymes are clearly important for DNA methylation in all cancers thus far studied.

## 6. On the Horizon

As discussed, the epigenome is comprised of multiple modifications to the genome. Certainly, DNA methylation, histone modifications, and miRs are key players, but the global transcriptome is affected by many other protein-protein and protein-nucleic acid interactions that in concert can effect changes through overall chromatin organization, chromosomal territory localizations, and gene-gene interactions through loops of inter- and intra-chromosomal DNA. The chromatin state is an epigenomic mechanism for wide-spread nuclear activities. For instance, the patterns of euchromatin and heterochromatin differ between lymphoma subtypes, as evidenced by electron microscopy. In general, the transcriptionally active euchromatic regions are found near the center of the nucleus, while less active heterochromatic regions generally localize near the nuclear membrane. Even beyond the visual differences, with the aid of a powerful methods such as Hi-C and formaldehyde-assisted isolation of regulatory elements (FAIRE) [85] coupled with genomic sequencing (FAIRE-seq) [86], it can been shown that the actual genomic regions and associated genes found in either the euchromatic or heterochromatic regions of the nucleus also differ by cell type. Job Dekker’s laboratory has developed multiple methods to probe genomic interactions. The most recent, Hi-C, is a method for defining the three-dimensional architecture of entire genomes through proximity-based ligation followed by massively parallel NGS. Through construction of spatial proximity maps of the human genome at a resolution of 1 megabase, they confirmed the presence of chromosome territories and the spatial proximity of small, gene-rich chromosomes, as well as an additional level of genome organization characterized by the spatial segregation of euchromatin and heterochromatin to form two genome-wide compartments [87]. Thus, Hi-C and NGS can be used to map the dynamic conformations of entire genomes. A complementary method, FAIRE-seq, can be used to investigate the genomic regions associated with the open chromatin regions of cells. Modifications to this method should also allow investigation of the protein-associated DNA found mainly in heterochromatic regions. It is inevitable that further refinements in NGS will facilitate studies at multiple levels of the epigenome and chromatin organization that can provide global information affecting the cellular transcriptome, proteome, and biological activity that defines clinical behaviors in all types of lymphoma, as well as other cancers and normal cells.
